# Rating scales for shoulder and elbow range of motion impairment: Call for a functional approach

**DOI:** 10.1371/journal.pone.0200710

**Published:** 2018-08-01

**Authors:** Anouk M. Oosterwijk, Marianne K. Nieuwenhuis, Hennie J. Schouten, Cees P. van der Schans, Leonora J. Mouton

**Affiliations:** 1 Research group Healthy Ageing, Allied Health Care and Nursing, Hanze University of Applied Sciences Groningen, Groningen, the Netherlands; 2 Association of Dutch Burn Centers, Burn Center Martini Hospital, Groningen, the Netherlands; 3 Department of Rehabilitation Medicine, University Medical Center Groningen, University of Groningen, Groningen, the Netherlands; 4 Center for Human Movement Sciences, University Medical Center Groningen, University of Groningen, Groningen, the Netherlands; 5 Department of Physiotherapy, Red Cross Hospital, Beverwijk, the Netherlands; 6 Department of Health Psychology, University Medical Center Groningen, University of Groningen, Groningen, the Netherlands; Augusta University, UNITED STATES

## Abstract

**Background:**

To evaluate the effect of (new) treatments or analyse prevalence and risk factors of contractures, rating scales are used based on joint range of motion. However, cut-off points for levels of severity vary between scales, and it seems unclear how cut-off points relate to function. The purpose of this study was to compare severity ratings of different rating scales for the shoulder and elbow and relate these with functional range of motion.

**Methods:**

Often used contracture severity rating scales in orthopedics, physiotherapy, and burns were included. Functional range of motion angles for the shoulder and elbow were derived from a recent synthesis published by our group. Shoulder flexion and elbow flexion range of motion data of patients three months after a burn injury were rated with each of the scales to illustrate the effects of differences in classifications. Secondly, the shoulder and elbow flexion range of motion angles were related to the required angles to perform over 50 different activities of daily living tasks.

**Results:**

Eighteen rating scales were included (shoulder: 6, elbow: 12). Large differences in the number of severity levels and the cut-off points between scales were determined. Rating the measured range of motions with the different scales showed substantial inconsistency in the number of joints without impairment (shoulder: 14–36%, elbow: 26–100%) or with severe impairment (shoulder: < 10%–29%, elbow 0%–17%). Cut-off points of most scales were not related to actual function in daily living.

**Conclusion:**

There is an urgent need for rating scales that express the severity of contractures in terms of loss of functionality. This study proposes a direction for a solution.

## Introduction

Many patients suffer from joint contractures as a secondary condition. These contractures include the shortening of muscle, tendon, ligament, or skin and can be a result of adhesive capsulitis, bone fractures, plexus lesions, cerebral palsy, rheumatoid arthritis, spinal cord injury, stroke, multiple sclerosis [1–11], and also aging [12]. Joint contractures are defined as a loss of range of motion (ROM) and may affect activities of daily living as well as participation and quality of life [13–17]. To evaluate the effect of (new) treatments or analyse prevalence and risk factors of contractures, many different rating scales are employed in orthopaedics and physiotherapy. A rating scale can include only the ROM in a specific direction of movement of a specific joint or can be combined with other dimensions such as pain and muscle force to yield a summarized value for describing the impact of the impaired joint on the patient [18].

Using scales to rate the severity of impaired ROM, however, is not without difficulties. First, the cut-off points for the levels of severity of different rating scales seem to vary which hampers comparing study results. Second, it seems to be ambiguous how the cut-off points of existing scales are related to function, a point that was also stressed in various earlier publications [17, 19–23]. In the present study, therefore, different rating scales for the shoulder and elbow were compared, and their severity ratings contrasted to functional ROM. Actual patient data, in this case patients with burns, were used to clarify issues.

## Methods

The most often utilized rating scales for assessing loss of ROM were selected based on reviews on the evaluation of shoulder and elbow function and/or rating scales [24–26]. In addition, contracture severity scales used for burns were included [17, 19, 27–28]. The cut-off points for shoulder (forward) flexion and elbow flexion ROM were extracted. In the event that a rating scale combined ROM with other dimensions such as pain and muscle force, only information pertaining to ROM was included.

Functional range of motion angles for shoulder and elbow flexion were derived from a recent synthesis of available data performed by our group [29–30]. Briefly, data from 36 studies involving a total of 66 ADL tasks were included (see for search strategy and outcomes Oosterwijk et al., 2018 [29–30]). In these studies, shoulder (flexion, extension, abduction, and adduction) and/or elbow (flexion, extension) angles had been measured in healthy subjects naturally performing ADL tasks, and angles were provided per movement direction and task. Angles for shoulder and elbow flexion are available from 53 tasks.

To facilitate comparison between the severity levels of scales and functional angles, rating scales were arranged chronologically and translated to figures whereby normal ROM for shoulder flexion was established at 0–180° and elbow flexion at 0–150° [11].

To illustrate the consequences of using different rating scales, range of motion data for shoulder (forward) flexion and elbow flexion of 39 patients three months after their burn injury were used. These data are part of a larger study in the Netherlands on contractures after burn injury. The study aim, design and procedures were discussed and approved by the research group of the Burn Center of the Martini Hospital Groningen. All procedures were in accordance with the ethical standards of the Helsinki declaration on ethical standards. The study protocol was reviewed by the Medical Ethical Committee (Martini Hospital Groningen no. 2011–19), which concluded no informed consent of patients was required, as the assessments concerned standard clinical practice. The patients included in the present study had been admitted to the burn centre of Groningen in 2011–2012 with burns across or adjoining a total of 63 shoulder(s) and/or elbow(s) (see S1 Table for patient and burn characteristics). The patient’s passive ROM was measured with a lateral goniometer (BaselineTM 12.5 inch, 3608 transparent plastic goniometer) according to the standardized protocols of Norkin and White [31]. Using these patient data, the severity of shoulder and elbow flexion impairment was determined by rating the measured ROM with each of the included rating scales. Secondly, to classify the functional consequences of impaired shoulder and elbow flexion, the ROM angle was related to functional angles per patient, i.e., to the angle required to perform ADL tasks.

## Results

### Rating scales

In total, 18 scales to rate the severity of impaired ROM were included; six for the shoulder [17, 19, 27–28, 32–34] and 12 for the elbow [17, 19, 27–28, 35–42] (Tables 1 and 2). Nine scales [32–34, 36–42] had additional items besides ROM to classify the severity of injury to the impaired joint (Tables 1 and 2).

**Table 1:** Shoulder flexion rating scales.

Reference	Year	Scale name	Abbreviated scale name	Entirely ROM based
Dobbs and Curreri [27]	1972	Dobbs burn contracture scale	Dobbs scale	Yes
Huang et al. [28]	1977	Huang burn contracture scale	Huang scale	Yes
Ellman et al. [32]	1986	UCLA shoulder rating scale ^a^	UCLA scale	No
Constant et al. [33–34]	1987/2008	Constant score	Constant score	No
Schneider et al. [19]	2006	Schneider burn contracture scale	Schneider scale	Yes
Niedzielski and Chapman [17]	2015	Burn Scar Contracture Severity Scale	BSC-SS	Yes

^a^ UCLA: University of California at Los Angeles

**Table 2:** Elbow flexion rating scales.

Reference	Year	Scale name	Abbreviated scale name	Entirely ROM based
Dobbs and Curreri [27]	1972	Dobbs burn contracture scale	Dobbs scale	Yes
Flynn et al. [35]	1974	Flynn Criteria	Flynn criteria	Yes
Ewald [36]	1975	Ewald scoring system	Ewald score	No
Huang et al. [28]	1977	Huang burn contracture scale	Huang scale	Yes
Inglis and Pellici [37]	1980	Hospital for Special Surgery scale	HSS	No
Morrey et al. [38]	1985	Mayo Elbow Performance Index	MEPI	No
Khalfayan et al. [39]	1992	Khalfayan scoring system	Khalfayan score	No
Morrey et al. [40]	1993	Mayo Elbow Performance Score	MEPS	No
Timmerman and Andrew [41]	1994	Timmerman-Andrew scoring system	T-A score	No
Sathyamoorthy et al. [42]	2004	Liverpool Elbow Score	LES	No
Schneider et al. [19]	2006	Schneider burn contracture scale	Schneider scale	Yes
Niedzielski and Chapman [17]	2015	Burn Scar Contracture Severity Scale	BSC-SS	Yes

#### Rating shoulder flexion

**Fig 1:**
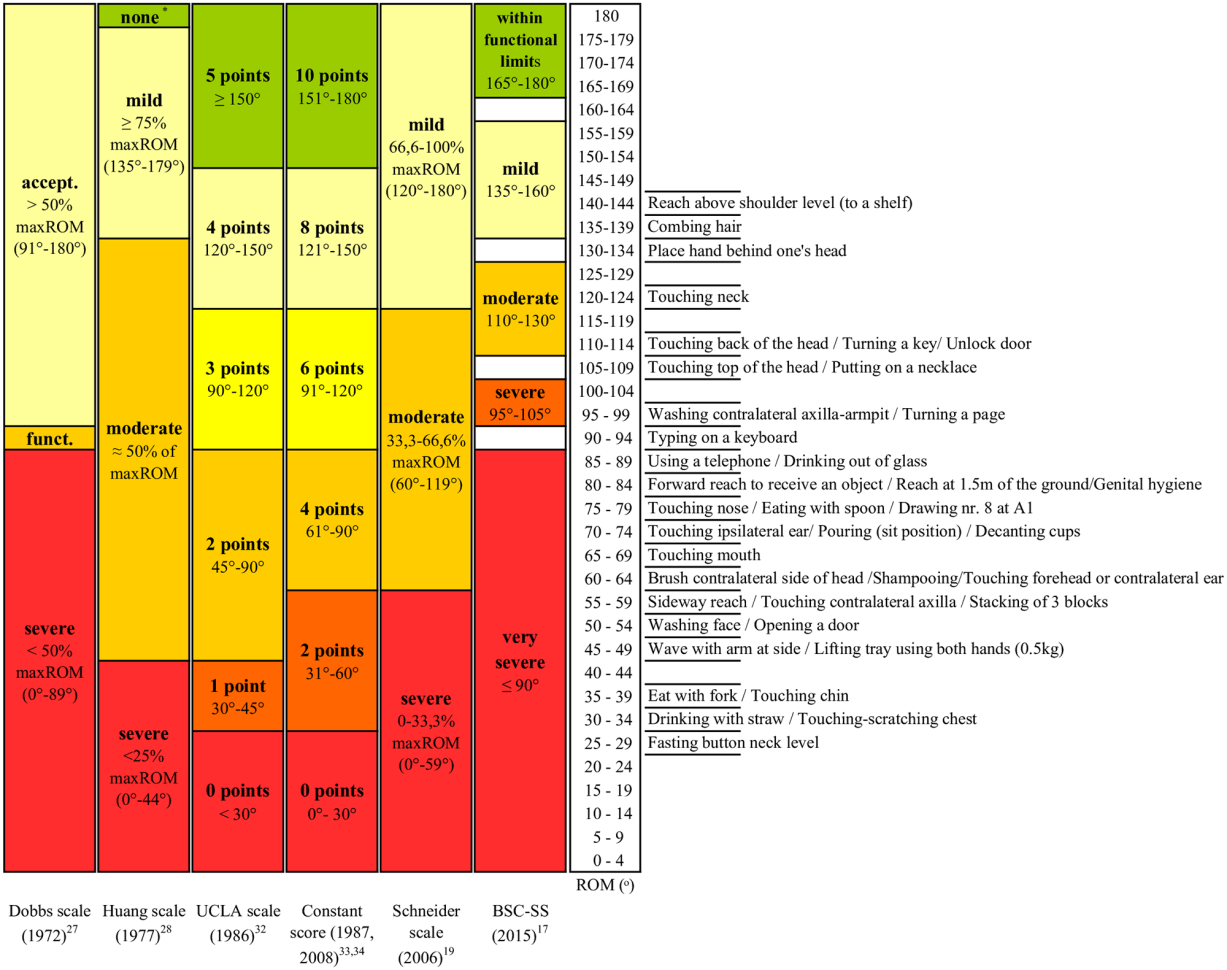
Schematic illustration of shoulder flexion rating scales and their cut-off points per degrees of Range of Motion (ROM) as well as required shoulder flexion ROM for activities of daily life (ADL) as synthesized by Oosterwijk et al. [29–30]. BSC-SS: Burn scar contracture severity scale; accept.: acceptable; funct.: functional = about 50% max ROM; *: none = no limitation of motion.

The six rating scales for shoulder flexion are shown in Fig 1. There were many differences in the numbers of levels and cut-off points between the levels. The Dobbs scale and the Schneider scale had fewest severity levels, i.e., three, while six levels were defined in the UCLA scale and the Constant score (Fig 1). The cut-off points of the Constant score, UCLA scale, and Schneider scale were rather similar; the only difference is the number of levels. Concerning BSC-SS, a number of degrees were not allocated to a severity rating. The degrees falling in between the levels were classified to the nearest level.

Four of the scales have a level for ‘no contracture’; in the Huang scale defined as ‘none’, in the BSC-SS defined as ‘within functional limits (WFL)’ and in the UCLA scale and Constant score receiving maximal points. The cut-off points of this ‘no contracture’ differed, i.e., 180° only, 151°-180°, ≥150°-180° and 165°-180° (Fig 1). The two other scales did not define a level for ‘no contracture’. The largest obvious difference in cut-off points is found at the (very) severe level with a much higher ROM angle (<90 and ≤90°) in the Dobbs scale and BSC-SS compared to the others.

In terms of function, as ascertain from the literature [29–30], shoulder flexion angles <25° were not required for any ADL task. Angles between 90° and 135° involved tasks for personal care whereby the hand needs to be placed on the upper body or head. Reaching above shoulder level (142°) was the task requiring the highest shoulder flexion angle. Comparing levels of severity to function (i.e., ROM angles required for ADL tasks), it was discovered that many tasks require angles in the middle range of the scales levels (Fig 1). If a contracture would prohibit performance of approximately 50% of these tasks, only the Dobbs scale and the BSC-SS would classify this as a severe contracture.

#### Rating elbow flexion

**Fig 2:**
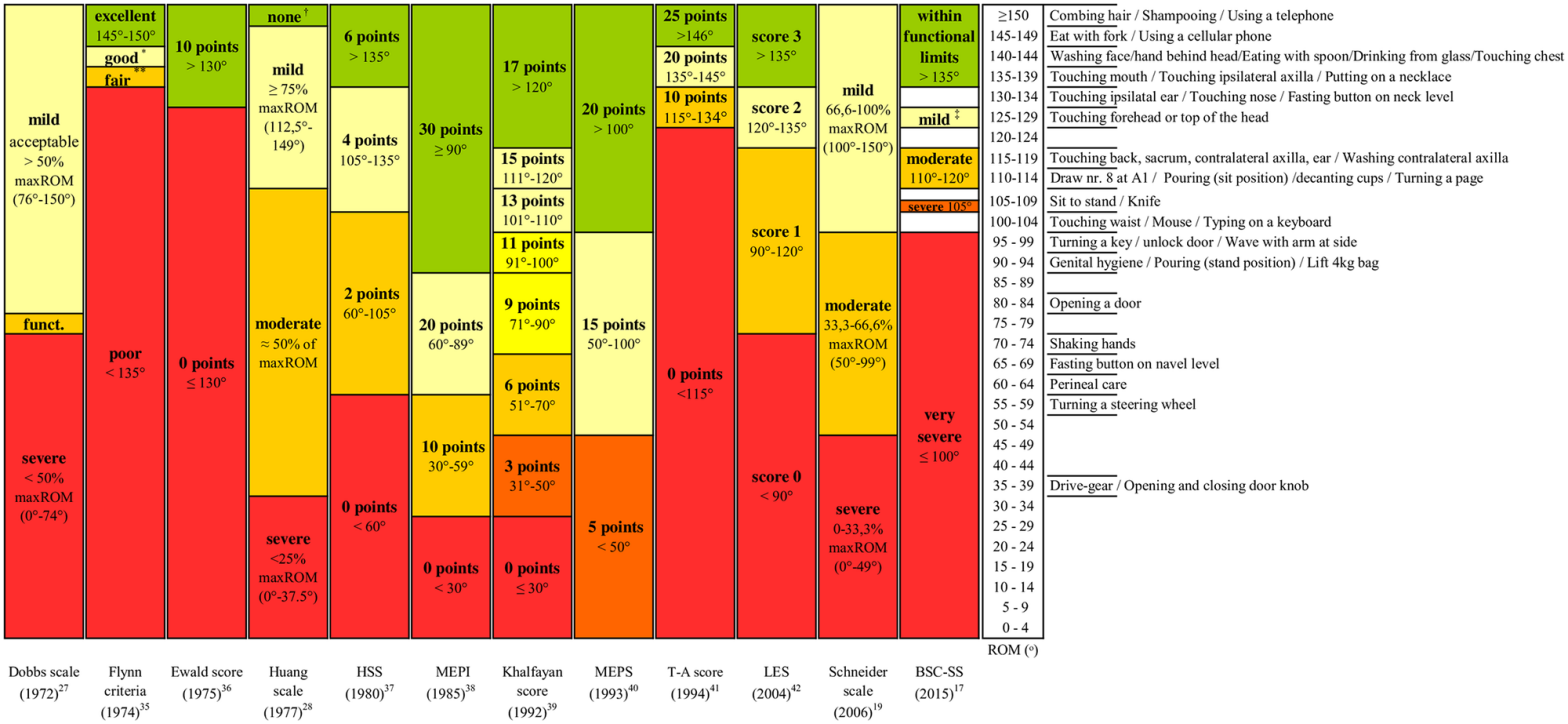
Schematic illustration of elbow flexion rating scales and their cut-off points per degrees of Range of Motion (ROM) as well as required elbow flexion ROM for activities of daily life (ADL) as synthesized by Oosterwijk et al. [29–30]. HSS: Hospital for special surgery scoring; MEPI: Mayo elbow performance index; MEPS: Mayo elbow performance score; T-A score: Timmerman-Andrew elbow score; LES: Liverpool elbow score; BSC-SS: Burn scar contracture severity scale; accept.: acceptable; funct.: functional = about 50% max ROM; *: good = 140°- 144°, ** fair = 135°- 139°, †: none = no limitation of motion, ‡: mild = 125°-130°.

The twelve contracture severity scales for elbow flexion are shown in Fig 2. The range in number of severity levels was substantial, i.e., from two in the Ewald score to eight in the Khalfayan score. All others described three to five levels. Ten of the 12 scales had a level for ‘no contracture’ with the cut-off points for these ranging from ≥90° to ≥150°. Almost all of the scales included a (very) severe level. The cut-off points for the most severe level of impairment also differed considerably between the scales, ranging from <30° to <135°. The MEPS still allocates five points with zero degrees of ROM.

In terms of function, performance of many ADL tasks required a high degree of elbow flexion with 16 of the 45 tasks needing a flexion angle of ≥135°. These tasks mainly comprised tasks required for personal care and feeding, although the largest angle required was determined for ‘using a telephone’ (152°) [29–30]. Comparing levels of severity to function (i.e., ROM angles required for ADL tasks), it was found that many tasks need angles located in the higher ranges of the scales’ levels (Fig 2). If a contracture prohibited performance of these tasks, there was a large difference in how the severity of contracture would be rated from no impairment to a severe impairment.

### ROM data applied to the contracture rating scales and expressed in terms of functionality

#### Shoulder flexion ROM data applied to contracture rating scales

To illustrate the implications of the different rating scales, actual patient data of 28 burned shoulders were used to rate shoulder flexion. The results of rating patient data in the contracture scales show that all levels of severity were found in all scales (Table 3). However, differences were discerned between scales. First, there was inconsistency in how many shoulders were rated as having ‘no’ shoulder flexion impairment (range 14–36%). Second, focussing on the other end of the scales, 29% of the shoulders were rated as being severely impaired based on the Dobbs scale and BSC-SS whereas all others scales classified less than 10% being severely impaired.

**Table 3:** Severity of shoulder flexion impairment according to six rating scales based on measured ROM of 28 shoulders three months post burn.

Dobbs scale		Huang scale		UCLA scale		Constant score		Schneider scale		BSC-SS	
Score	%	Score	%	Pts	%	Pts	%	Score	%	Score (pts)	%
Acceptable	71%	None	14%	5	36%	10	36%	Mild	64%	WFL (0)	18%
Functional	0%	Mild	43%	4	25%	8	25%	Moderate	29%	Mild (1)	43%
Severe	29%	Moderate	36%	3	11%	6	11%	Severe	7%	Moderate (2)	4%
		Severe	7%	2	21%	4	21%			Severe (3)	7%
				1	4%	2	4%			Very severe (4)	29%
				0	4%	0	4%				

#### Shoulder flexion ROM expressed in terms of functionality

**Fig 3:**
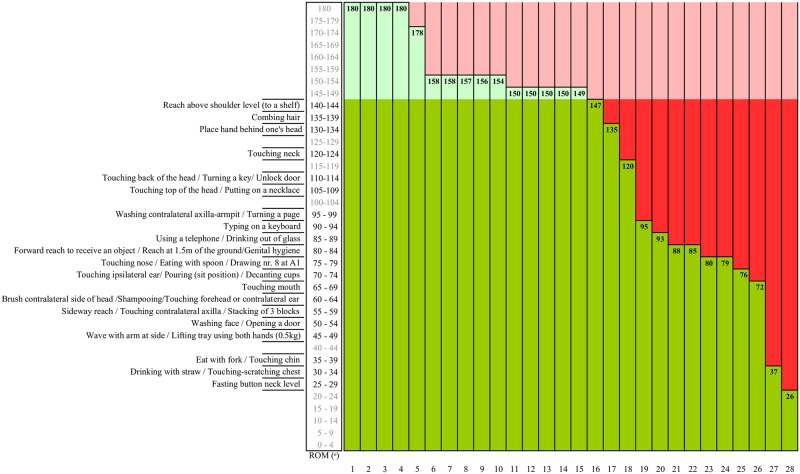
Schematic illustration of activities of daily life (ADL) that can (dark green) or cannot (dark red) be performed after burn injury in the shoulder joint area, based on measured Range of Motion (ROM) of 28 shoulders 3 month post burn (ROM given in bold numbers) and the required shoulder flexion ROM for 36 different ADL tasks as synthesized by Oosterwijk et al. [29–30]. Light green and light red represent joint angles that can or cannot be performed, respectively, but do not affect functionality based on the included tasks. Note that not many of the tasks need near full (145°-180°) shoulder flexion ROM meaning that up to 35° ROM deficit might have rather limited impact on daily functioning.

Regarding the functional consequences, patients would be able to perform all ADL tasks with the ROM as measured in 57% (16/28) of the burned shoulders (Fig 3). The limitations in two shoulders (7%) would cause problems only in performing high reaching activities, combing hair, and touching the neck. The other ten (37%) burned shoulders would cause severe functional limitations with two shoulders causing very severe limitations (Fig 3).

The scales in which the cut-off value for severe contractures best compares to function are the Dobbs scale and the BCS-SS. The Dobbs scale classified 29% and the BSC-SS classified 36% of the shoulders as (very) severely limited. The upper two levels of the UCLA scale, Constant score, and BSC-SS would rate 61% of the shoulders as having no or little contracture which is nearest to the 57% having no functional problems, i.e., able to perform all ADL tasks.

#### Elbow flexion ROM data applied to contracture rating scales

Scoring elbow flexion data of 35 burned elbows with the twelve elbow rating scales showed that, in four scales (Dobbs scale, MEPI, MEPS, Schneider scale), all elbows scored on only one severity level (Table 4). In the HSS, Khalfayan score, and BSC-SS, all elbows scored in the upper levels but not in the lower levels of their scales.

**Table 4:** Severity of elbow flexion impairment according to 12 rating scales based on measured ROM of 35 elbows three months post burn.

**Dobbs scale**		**Flynn Criteria**		**Ewald Score**		**Huang scale**	
Score	%	Score	%	Pts	%	Score	%
Acceptable	100%	Excellent	49%	10	86%	None	26%
Functional	0%	Good	23%	0	14%	Mild	54%
Severe	0%	Fair	11%			Moderate	20%
		Poor	17%			Severe	0%
**HSS**		**MEPI**		**Khalfayan score**		**MEPS**	
Pts	%	Pts	%	Pts	%	Pts	%
6	80%	30	100%	17	91%	20	100%
4	20%	20	0%	15	6%	15	0%
2	0%	10	0%	13	3%	5	0%
0	0%	0	0%	11	0%		
				9	0%		
				7	0%		
				5	0%		
				3	0%		
				0	0%		
**T-A score**		**LES**		**Schneider scale**		**BSC-SS**	
Pts	%	Pts	%	Score	%	Score (pts)	%
25	31%	3	80%	Mild	100%	WFL (0)	83%
20	51%	2	11%	Moderate	0%	Mild (1)	9%
10	11%	1	9%	Severe	0%	Moderate (2)	9%
0	6%	0	0%			Severe (3)	0%
						Very severe (4)	0%

Concerning ‘no contracture’, even larger differences were found for the elbow than for the shoulder, i.e., the percentage of elbows that scored maximally ranged from 100% (Dobbs scale, MEPI, MEPS, Schneider scale) to 26% (Huang scale). Elbow flexion angles corresponding to the most severe level were only determined by employing the Flynn criteria (17% scored poor), Ewald score (14% with 0 points), and T-A score (6% with 0 points).

#### Elbow flexion ROM data expressed in terms of functionality

**Fig 4:**
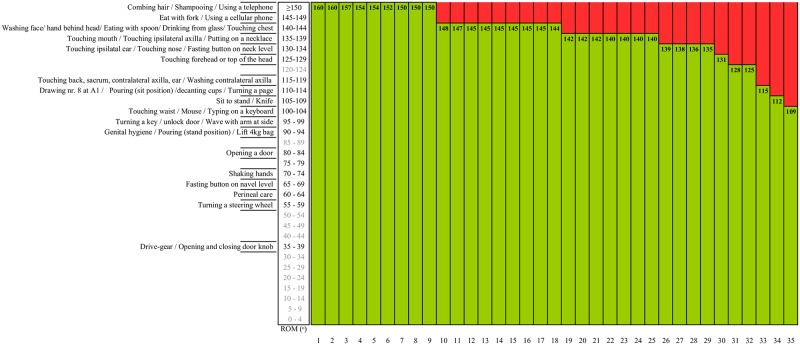
Schematic illustration of activities of daily life (ADL) that can (green) or cannot (red) be performed after burn injury in the elbow joint area, based on measured Range of Motion (ROM) of 35 elbows 3 month post burn (ROM given in bold numbers) and the required elbow flexion ROM for 44 different ADL tasks as synthesized by Oosterwijk et al. [29–30]. Note that many of these ADL tasks need almost full elbow flexion so that even a small ROM deficit can have considerable impact on daily functioning.

Many of the ADL tasks required almost full elbow flexion so that even a small ROM deficit had considerable impact on ADL. Regarding functional consequences, with the ROM as measured in 26% (9/35) of the elbows, patients would be able to perform all ADL tasks. All other elbows (74%) would be more or less severely impaired (Fig 4). Comparing severity levels with the limitation in ADL functioning for elbow flexion, all rating scales underrate the impact of limitations in ROM of elbow flexion on daily functioning.

## Discussion

In the present study, different rating scales for the shoulder and the elbow were compared, and their severity ratings contrasted with functional ROM. Large differences in the number of severity levels and angles corresponding to cut-off points between scales were determined. Rating the measured ROMs with the different scales demonstrated substantial inconsistency in how many joints were classified as having no contracture (shoulder: 14–36%, elbow: 0–100%) and, at the other end of the spectrum, a severe contracture (shoulder: 4%–29%, elbow 26%–100%). As indicated before, there is an urgent need for scales that express the severity of contractures in terms of function. The present review emphasizes this by showing that cut-off points of most included scales were not related to function. When comparing severity levels with the limitation in ADL functioning for elbow flexion, all rating scales underrated the impact on daily functioning.

Concerning the used scales, the lower part of the Dobbs scale and BSC-SS and the upper part of the Constant score, UCLA scale, and BSC-SS seem most in accordance with shoulder flexion function. For elbow flexion, the Flynn criteria is most in line with function because of the extensive level of ‘severe’ that is included in this scale. Considering the available data, we suggest an upper cut-off point of 145° and lower cut-off point of approximately 95° for the shoulder flexion whereby an active shoulder flexion of more than 145° corresponds to no functional contracture and less than 95° of active shoulder flexion corresponds to a (very) severe functional contracture. For elbow flexion, we suggest an upper cut-off point of 150° with >150° meaning ‘no contracture’ or ‘no functional limitations’ and a lower cut-off point of 140° with <140° meaning a contracture with (very) severe functional consequences. Discussion is open for the ROM in between and the number of levels whereby levels are based preferably on clinically minimally important differences and taking into account imprecision of assessment especially when goniometry is used [43].

Concerning the distribution of tasks over the total range of motion per joint [29–30], it is clear that a rating scale with the same cut-off points for all joints cannot be viable. Even the various movement directions of the same joint have different distributions of tasks over the total range of motion. Therefore, for each joint and movement direction, a specific functional scale should be developed.

### Limitations

First, we did not perform a systematic literature review to unearth all of the rating scales but used the scales that are commonly utilized as evidenced from review articles. In this aspect, we think we have included the most obvious and relevant rating scales. Second, angles required for functional range of motion were based on the information of all tasks that were available from the literature, i.e., from a total of 53 ADL tasks for shoulder flexion and elbow flexion. This information on available tasks, however, does not cover all daily activities, for example, dressing tasks could not be included as they have not yet been assessed. This may be explained by the fact that putting on clothes would cover markers necessary for assessment. When such data becomes available, interpretation including the differentiation of cut-off points may change. Furthermore, the required ROM per ADL task ascertain in the review [29–30] were based on active ROM whereas the data from patients with burns were passive ROM; though, in our opinion, this does not change the conclusions of this study. Finally, task execution can be influenced by age, gender, hand dominance, and/or a postural or upper limb length variability [44–48] and, therefore, the functional angles will not be representative for each individual.

The relevance of the range of motion of shoulder and elbow joints for an individual is more than can be covered by ADL tasks. Depending on individual wishes and demands on the mobility of these joints concerning, for example, work and leisure time activities, the ROM angles that are needed may vary. Furthermore, during task execution, multiple joints move together in a chain. Therapists will have to keep this in mind in their treatment of individual patients. When comparing the effectiveness of different treatment strategies or evaluating prevalence and risks factors, the functional relevance of ROM angles in terms of ADL as a universal demand on ROM is a good starting point.

### Further research

To derive a more functional scale to rate the severity of contractures, further research should focus on expanding the amount and diversity of tasks (including, for example, dressing tasks) and being aware of the differences of participants’ characteristics. Furthermore, to optimize and tailor interventions to maintain or improve mobility, additional research is required on the correlation between objective ROM impairment and problems in ADL as well as participation and quality of life as experienced by patients. In reality, it is possible that a ROM impairment is not considered a problem as the patient is able to perform all activities with compensatory movements (i.e., using surrounding joints or the other arm). However, compensatory movements can lead to serious secondary conditions such as overuse of muscles around the affected joint, an increased risk of soft tissue problems and degenerative joint diseases [49–51]. In this regard, it would not only be relevant to know which ROM angles are required but also how often extreme ROMs are used during the course of a day. Maintaining or restoring ROM to be able to naturally perform ADL tasks is crucial whereas evaluation of compensatory movements should be a focus for further research. Finally, we have made a start in the functional approach for shoulder and elbow flexion, but more research is necessary for other joints and movement directions in healthy and impaired participants.

## Conclusion

The use of various different classifications for the shoulder and elbow obscures the true impact of contractures and, therefore, hampers clinical practice as well as research. There is an urgent need for rating scales expressing the severity of contractures in terms of loss of function. This study provides some solution indications, but much work is still needed. We hope to have encouraged discussion and further research.

## Supporting information

S1 Table Demographic and medical characteristics of the study population. (PDF)
