# Supplementary material for: Rating scales for shoulder and elbow range of motion impairment: Call for a functional approach
Source: PLoS One. 2018 Aug 1;13(8):e0200710. doi: 10.1371/journal.pone.0200710 (PMC6070201; doi:10.1371/journal.pone.0200710)
Supplement: S1 Table — (PDF) [file pone.0200710.s001.pdf]

Supporting information Table 1: Demographic and medical characteristics of the study population.

		Total[#]	Shoulder	Elbow
N subjects		39	20	26
N burned shoulder/ elbow		63	28	35
Location burn injury	Left side	-	4	4
	Right side	-	8	13
	Bilateral	-	8	9
Sex (% male)		62	55	69
Age (mean (sd) in years)		37.8 (23.0)	43.0 (24.3)	34.3 (22.1)
Age (range in years)		0-79	0-79	0-65
TBSA (mean (sd) in %)		14.8 (16.2)	17.2 (14.4)	15.8 (17.2)
TBSA (range in %)		-	1-48%	1-66%
Full thickness TBSA (mean (sd) %)		3.5 (8.9)	4.5 (8.5)	4.7 (10.5)
LOS (mean (sd) days)		29.8 (22.4)	34.4 (18.2)	30.3 (25.6)

[#] In some patients more than one joint is burned; TBSA: Total Body Surface Area; LOS: Length of stay at the hospital.
